# A Novel Survival-Based Tissue Microarray of Pancreatic Cancer Validates MUC1 and Mesothelin as Biomarkers

**DOI:** 10.1371/journal.pone.0040157

**Published:** 2012-07-06

**Authors:** Jordan M. Winter, Laura H. Tang, David S. Klimstra, Murray F. Brennan, Jonathan R. Brody, Flavio G. Rocha, Xiaoyu Jia, Li-Xuan Qin, Michael I. D’Angelica, Ronald P. DeMatteo, Yuman Fong, William R. Jarnagin, Eileen M. O’Reilly, Peter J. Allen

**Affiliations:** 1 Department of Surgery, Thomas Jefferson University, Philadelphia, Pennsylvania, United States of America; 2 Department of Pathology, Memorial Sloan-Kettering Cancer Center, New York, New York, United States of America; 3 Department of Surgery, Memorial Sloan-Kettering Cancer Center, New York, New York, United States of America; 4 Department of Surgery, Virginia Mason Medical Center, Seattle, Washington, United States of America; 5 Department of Epidemiology and Biostatistics, Memorial Sloan-Kettering Cancer Center, New York, New York, United States of America; 6 Department of Medicine, Memorial Sloan-Kettering Cancer Center, New York, New York, United States of America; Aix-Marseille University, France

## Abstract

**Background:**

One–fifth of patients with seemingly ‘curable’ pancreatic ductal adenocarcinoma (PDA) experience an early recurrence and death, receiving no definable benefit from a major operation. Some patients with advanced stage tumors are deemed ‘unresectable’ by conventional staging criteria (e.g. liver metastasis), yet progress slowly. Effective biomarkers that stratify PDA based on biologic behavior are needed. To help researchers sort through the maze of biomarker data, a compendium of ∼2500 published candidate biomarkers in PDA was compiled (PLoS Med, 2009. 6(4) p. e1000046).

**Methods and Findings:**

Building on this compendium, we constructed a *survival tissue microarray* (termed s-TMA) comprised of short-term (cancer-specific death <12 months, n = 58) and long-term survivors (>30 months, n = 79) who underwent resection for PDA (total, n = 137). The s-TMA functions as a biological filter to identify bona fide prognostic markers associated with survival group extremes (at least 18 months separate survival groups). Based on a stringent selection process, 13 putative PDA biomarkers were identified from the public biomarker repository. Candidates were tested against the s-TMA by immunohistochemistry to identify the best markers of tumor biology. In a multivariate model, MUC1 (odds ratio, OR = 28.95, 3+ vs. negative expression, p = 0.004) and MSLN (OR = 12.47, 3+ vs. negative expression, p = 0.01) were highly predictive of early cancer-specific death. By comparison, pathologic factors (size, lymph node metastases, resection margin status, and grade) had ORs below three, and none reached statistical significance. ROC curves were used to compare the four pathologic prognostic features (ROC area = 0.70) to three univariate molecular predictors (MUC1, MSLN, MUC2) of survival group (ROC area = 0.80, p = 0.07).

**Conclusions:**

MUC1 and MSLN were superior to pathologic features and other putative biomarkers as predicting survival group. Molecular assays comparing cancers from short and long survivors are an effective strategy to screen biomarkers and prioritize candidate cancer genes for diagnostic and therapeutic studies.

## Introduction

While pancreatic ductal adenocarcinoma (PDA) is typically aggressive as compared to most other cancers, the disease is comprised of a range of biological phenotypes. Roughly 20% of patients who undergo resection will live at least 5 years, and a similar percentage of patients will recur early after resection and die of disease within a year [1]–[4]. At the genomic level, each PDA acquires a unique constellation of somatic mutations [5]. Molecular diversity at the RNA and protein levels is even more complex. Despite the genotypic and phenotypic diversity in PDA, there are no reliable or clinically relevant prognostic biomarkers that stratify the disease based on predicted outcome.

Pathology reports include basic information regarding the stage and grade of the tumor, and currently provide the best available prognostic information. Conventional pathologic features remain the prognostic gold standard (e.g. lymph node status and histologic grade). However, across multiple large studies, adjusted hazard ratios for pathologic features are below two [6]–[8]. Similarly, in a validated pancreatic cancer nomogram, adverse pathologic features contribute less than 10% to 3-year survival predictions [9]. Serum CA19-9 is equally limited as a prognostic marker [10]–[12]. Prognostic information with such minimal predictive value cannot reliably inform treatment decisions. Furthermore, a complete set of pathologic data is only available for patients with resected cancers, which comprise a minority of patients with PDA.

Improved prognostic information is a priority of cancer research. First, accurate prognosis informs discussions between oncologists and patients about the natural history of pancreatic cancer. Second, the information can guide treatment decisions with implications for both quality of life and cancer-related outcomes. The most biologically aggressive PDAs (such as those that recur soon after resection) are best treated initially with systemic therapy, as opposed to major surgery. Pancreatic surgery delays systemic treatment by a minimum of 2 months and exposes the patient to substantial operative risk with little expected benefit. On the other hand, patients with indolent cancers with oligometastatic disease may benefit from an aggressive surgical approach, as has become standard of care in selected patients with metastatic colorectal cancer [13]. Third, prognostic biomarkers provide mechanistic insights into cancer development. Fourth, they serve as molecular targets for novel treatment strategies such as vaccine [14], antibody [15], and promoter-driven gene therapies [16].

High impact studies based on hundreds of patient samples have improved prognostic capabilities in multiple cancer types (e.g. lung, prostate, colon, and breast) [17]–[20]. Studies of similar magnitude and scope have proven difficult in pancreatic cancer due to less available tissue for study and less biological heterogeneity between tumors. Perhaps the most informative prognostic study to date in PDA identified a panel of 6 prognostic markers based on gene expression differences between localized PDA and autopsy specimens (n = 30) [21]. The rationale behind the study design was that the two study groups represented different ends of PDA extremes. In fact, the groups were actually distinguished by disease stage (i.e. early vs late), as opposed to biologic behavior (i.e. aggressive vs indolent). The localized group actually had a median survival of just 9 months, which is considered a short survival period post-resection [6].

**Figure 1:**
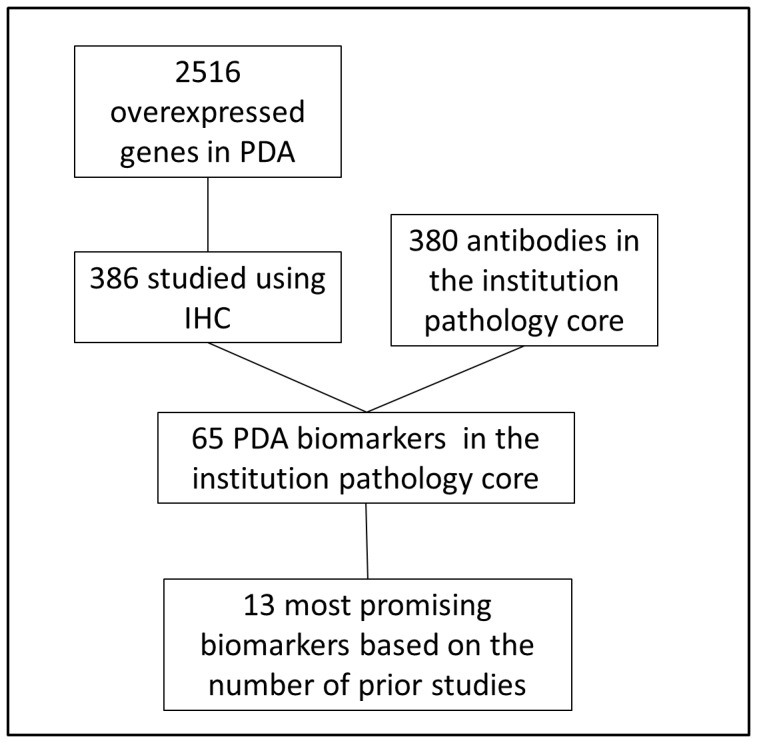
Algorithm for the selection of candidate biomarkers from a large public dataset of pancreatic cancer biomarkers [**22**].

In the present study, we used immunohistochemistry to interrogate a dichotomous set of resected PDAs (n = 137) comprised exclusively of aggressive (cancer-specific survival <12 months) and relatively less aggressive (cancer-specific survival >30 months) cancers, for true predictors of survival. A panel of 13 promising PDA biomarkers was selected from literally thousands of published PDA candidate biomarkers using a rigorous selection strategy (described in detail below), from on a public compendium of PDA biomarkers (Figure 1) [22]. Using this approach, we discounted 11 putative PDA biomarkers as prognostic markers. However, two proteins, mesothelin (MSLN) and mucin 1, cell surface associated (MUC1), were robust predictors of survival group and surpassed conventional pathologic features as prognostic factors. In this study, we demonstrated the utility of a large-scale, high throughput immunohistochemistry (IHC) based-assay of PDAs at survival extremes to identify bona fide biomarkers of aggressive cancer biology.

**Table 1:** Clinicopathologic features in short- (<12 months) and long-term survivors (>30 months).

Variable	Total N = 137, N (%)	Short survivors N = 58, N (%)	Long survivors N = 79, N (%)	P value
Lymph nodes				
Negative	38 (28%)	10 (17%)	28 (35%)	0.02
Positive	99 (72%)	48 (83%)	51 (65%)	
Hisotologic grade				
Well/Moderate	93 (68%)	31 (54%)	62 (78%)	0.003
Poor	44 (32%)	27 (47%)	17 (22%)	
Tumor size				
<3 cm	48 (36%)	14 (24%)	34 (44%)	0.02
≥3 cm	87 (64%)	44 (76%)	43 (56%)	
Resection margin				
Negative	116 (85%)	48 (83%)	68 (86%)	0.6
Positive	21 (15%)	10 (17%)	11 (14%)	
Age				
<70 years	72 (53%)	28 (48%)	44 (56%)	0.5
≥70 years	65 (48%)	30 (52%)	35 (44%)	
Gender				
Male	76 (55%)	30 (52%)	46 (58%)	0.5
Female	61 (45%)	28 (48%)	33 (42%)	
Length of stay (days), Median (range)	9 (4–40)	9 (5–35)	9.5 (4–40)	0.8
Adjuvant treatment				
No	51 (39%)	26 (47%)	25 (32%)	0.1
Yes	81 (61%)	29 (53%)	52 (68%)	

## Methods

### Patients

This study was approved by the Memorial Sloan-Kettering Cancer Center (MSKCC) institution review board. Patients were included if they underwent a pancreatic resection for invasive tubular type (conventional) ductal adenocarcinoma (PDA) after the year 2000, and either died of disease within 1 year of resection (short survival) or survived at least 30 months (long survival). The specific survival boundaries were chosen for two reasons: first, to yield groups that were sufficiently powered for the analysis, yet had comparable sample sizes to each other; second, so that that the time interval between the two survival groups (at least 1.5 years in the present study) emphasized tumor biology over treatment related determinants of survival. For instance, adjuvant treatment provides a survival benefit of roughly 3 months for PDA [23], and therefore should not dictate survival groups as defined here, except in rare cases. Similarly, recovery rates from surgery are variable, but patients who survive pancreatic resection generally return to their preoperative baseline, or suffer from disease-related symptoms [24]. The records of each patient in the short-term survival group were meticulously reviewed, and only patients who died from pancreatic cancer (and not complications from surgery) were included in the study. Patients with invasive cancer arising from an intraductal papillary mucinous neoplasm, colloid carcinoma, acinar cell carcinoma, and other less common variants of adenocarcinoma were excluded.

**Table 2:** IHC analysis in short- (<12 months) and long-term survivors (>30 months).

Biomarker, symbol	IHC Score	Total N = 137,N (%)		Short survivorsN = 58, N (%)	Long survivorsN = 79, N (%)	P value
BCL2	Negative	137 (100%)		58 (100%)	79 (100%)	–
CASP3	0	85 (62%)		35 (60%)	50 (63%)	0.67
	1+	32 (23%)		16 (28%)	16 (20%)	
	2+	18 (13%)		6 (10%)	12 (15%)	
	3+	2 (1%)		1 (2%)	1 (1%)	
CCND1	0	17 (12%)		8 (14%)	9 (11%)	0.43
	1+	51 (37%)		21 (36%)	30 (38%)	
	2+	60 (44%)		23 (40%)	37 (47%)	
	3+	9 (7%)		6 (11%)	3 (4%)	
CEACAM6	0	14 (10%)		7 (12%)	7 (9%)	0.69
	1+	8 (6%)		2 (3%)	6 (8%)	
	2+	12 (9%)		6 (10%)	6 (8%)	
	3+	103 (75%)		43 (74%)	60 (76%)	
EGFR	0	56 (41%)		24 (41%)	32 (41%)	1.0
	1+	38 (28%)		16 (28%)	22 (29%)	
	2+	38 (28%)		16 (28%)	22 (29%)	
	3+	5 (4%)		2 (3%)	3 (4%)	
ERBB2	0	111 (81%)		50 (86%)	61 (79%)	0.26
	1+	23 (17%)		8 (14%)	15 (19%)	
	2+	3 (2%)		0 (0)	3 (4%)	
MSLN	0	40 (29%)		8 (14%)	32 (41%)	<0.0001
	1+	31 (23%)		11 (19%)	20 (25%)	
	2+	45 (33%)		22 (38%)	23 (29%)	
	3+	21 (15%)		17 (29%)	4 (5)	
MUC1	0	20 (15%)		1 (2%)	19 (24%)	<0.0001
	1+	31 (23%)		9 (16%)	22 (28%)	
	2+	45 (33%)		20 (34%)	25 (32%)	
	3+	41 (30%)		28 (48%)	13 (16%)	
MUC2	Negative	116 (85%)		54 (93%)	62 (78%)	0.03
	Positive	21 (15%)		4 (7%)	17 (22%)	
MUC4	0	62 (45%)		23 (40%)	39 (49%)	0.70
	1+	30 (22%)		14 (24%)	16 (20%)	
	2+	18 (13%)		9 (16%)	9 (11%)	
	3+	27 (20%)		12 (21%)	15 (19%)	
MYC	0	51 (37%)		25 (43%)	26 (33%)	0.32
	1+	42 (31%)		19 (33%)	23 (29%)	
	2+	38 (28%)		13 (22%)	25 (32%)	
	3+	6 (4%)		1 (2%)	5 (6%)	
SMAD4	Negative	43 (31%)	23 (40%)		20 (25%)	0.09
	Positive	94 (69%)	35 (60%)		59 (75%)	
TP53	0	57 (42%)	22 (38%)		35 (44%)	0.21
	1+	17 (12%)	5 (9%)		12 (15%)	
	2+	35 (26%)	20 (34%)		15 (19%)	
	3+	28 (20%)	11 (19%)		17 (22%)	

Approved gene names are listed.

Percentages reflect the fraction in a given column.

B-Cell CLL/Lymphoma 2; Caspase 3; Cyclin D1; Carcinoembryonic antigen-related cell adhesion molecule 6 (non-specific cross reacting antigen); Epidermal Growth Factor Receptor; V-erb-b2 avian erythroblastic leukemia viral oncogene homolog 2; Mesothelin; Mucin 1, cell surface associated; Mucin 2, oligomeric mucus/gel-forming; Mucin 4, cell surface associated; V-myc avian myelocytomatosis viral oncogene homolog; Mothers against decapentaplegic, drosophila, homolog of, 4; Tumor protein p53.

**Figure 2:**
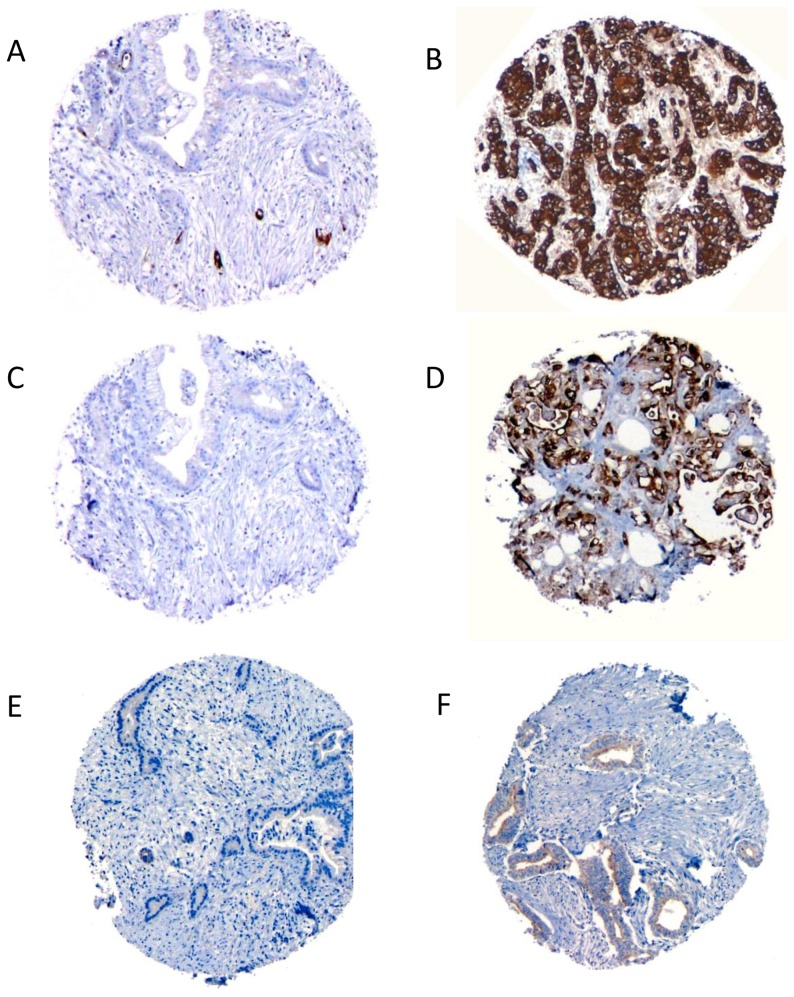
Representative immunolabeled slides: A) MUC1, 0; B) MUC1, 3+; C) MSLN, 0; D) MSLN, 3+; E) MUC2, negative; F) MUC2, positive.

### Clinicopathologic Information

Clinicopathologic information was extracted from the institutional pancreatic tumor database and from electronic patient records. Relevant clinical variables included postoperative chemotherapy, radiation therapy, and patient survival. Pathologic data included lymph node status (positive vs. negative), tumor differentiation (poor vs. moderate/well), size (≥3 cm vs. <3 cm), and resection margin status (positive vs. negative). Microscopic disease at the pancreatic neck, bile duct, duodenum, and uncinate margins were categorized as positive.

**Figure 3:**
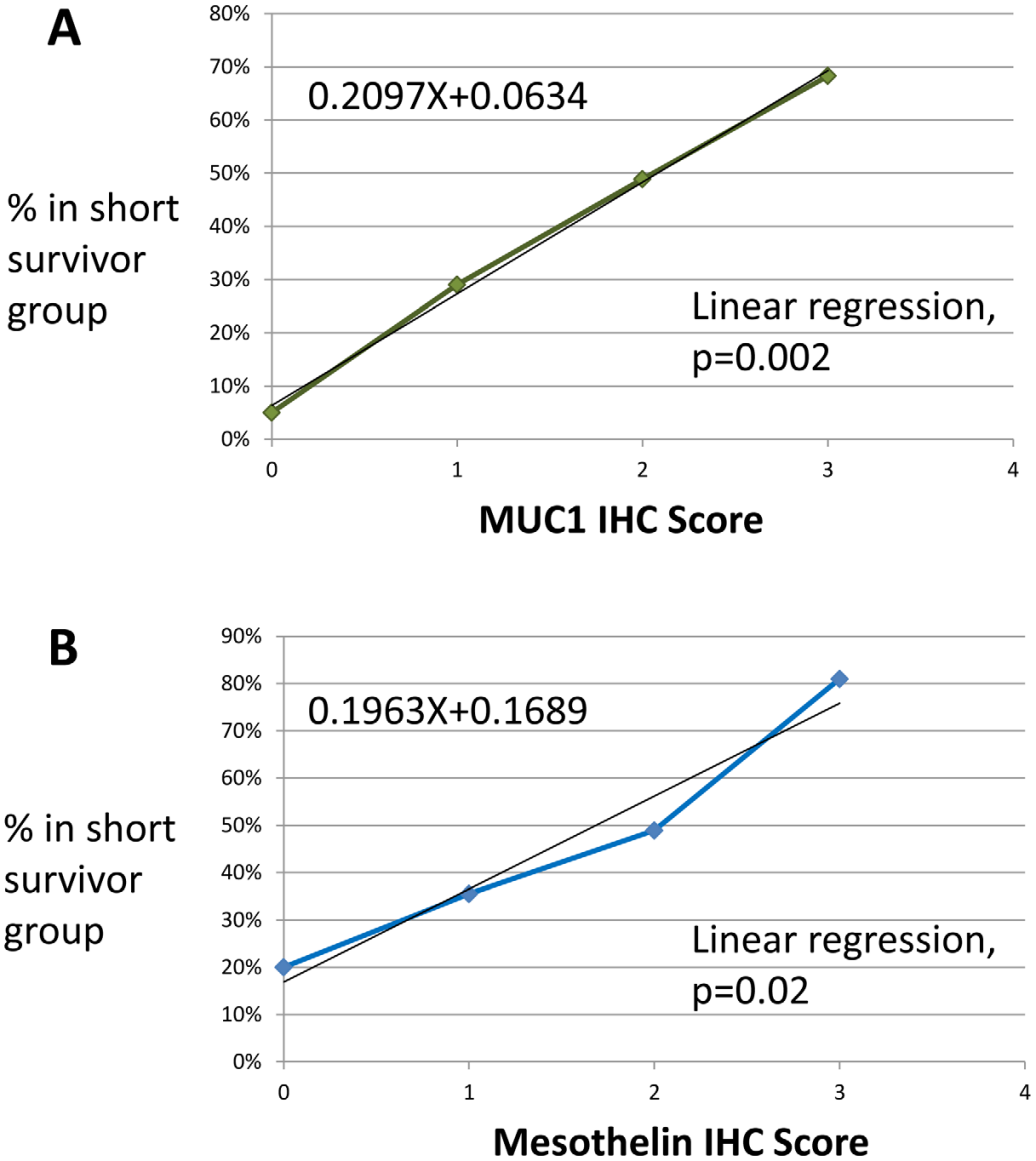
Short survivors (% of total) vs IHC score: A) MUC1 B) MSLN.

**Table 3:** Multivariate predictors of short-term survival.

Prognostic marker	OR	95% CI	P value
MSLN negative	Ref		0.01
MSLN 1+	1.65	(0.48, 5.72)	
MSLN 2+	2.64	(0.85, 8.22)	
MSLN 3+	12.47	(2.43, 64.14)	
MUC2 negative	Ref		0.72
MUC2 1+	0.77	(0.18, 3.32)	
MUC1 negative	Ref		0.004
MUC1 1+	10.12	(1.05, 97.50)	
MUC1 2+	11.91	(1.30, 108.91)	
MUC1 3+	28.95	(2.93, 285.64)	
Positive lymph node	2.79	(1.0, 7.83)	0.051
Poor differentiation	2.22	(0.84, 5.88)	0.11
Size≥3 cm	2.22	(0.89, 5.52)	0.09
Positive resection margin	2.36	(0.71, 7.85)	0.16

### Tissue Preparation

The TMA was constructed from tissue cores obtained from formalin-fixed, paraffin embedded tissue blocks in 151 patient samples. In all cases, tissue samples were derived from resected primary ductal adenocarcinomas of the pancreas. The technician placed the samples on the TMA in a blinded fashion ensuring that IHC interpretation by the study investigators was unbiased. The TMA was constructed as follows: a representative block of tumor was obtained and a corresponding H & E stained slide was examined under a microscope for foci of high neoplastic cellularity. Triplicate cores were taken from the index blocks and transferred to a virgin block for TMA processing with an automated tissue array machine (ATA-27, Beecher Instruments, Silver Spring, MD). TMA sections were then cut from the block in preparation for immunohistochemistry experiments.

**Figure 4.**
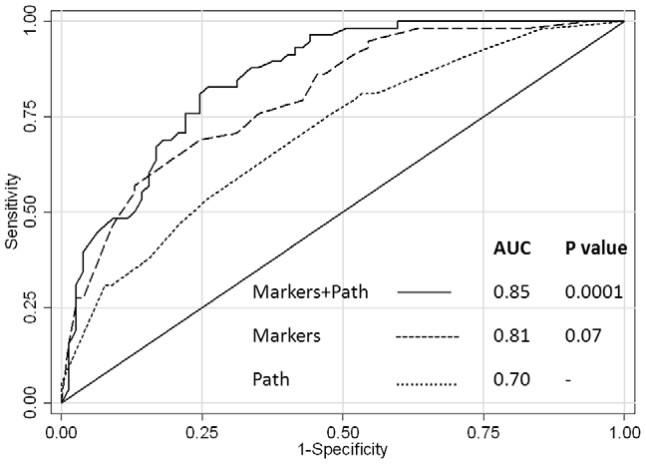
ROC curves of 3 predictive models of survival for the study cohort: Protein biomarkers and conventional pathologic features (——, MUC1, MUC2, MSLN, lymph node status, resection margin status, tumor differentiation); protein biomarkers only (------, MUC1, MUC2, MSLN); conventional pathologic features only (……, lymph node status, resection margin status, tumor differentiation, size). Values along the indicated diagonal line (*line of no-discrimination*) reflect a random guess, with points above the line being better than random. Harrel’s C-index or area under the curve (AUC) for each plot is provided. P values refer to comparisons between the given ROC curve as compared to pathologic features only.

**Table 4:** Published tissue microarrays with pancreatic cancer.

1^st^ Author	Institution	Sample Size	# Abs tested	Biomarker identified (Approved name)
1) Cao [47]	JHH	223	1	SERPINB5
2) Karamitopoulou [48]	Athens	210	4	CDKN1B and TP53
3) Yu [49], [50]	Shanghai	167	4	ATM, TP53, CDKN1A, MDM2
4) Tanaka [51]	Tokyo	156	1	CLDN18
**5) Present study** *	**MSKCC**	**137**	**13**	**MSLN and MUC1**
6) Chen [52]	Washington	127	2	ITGB1 and ANXA2
7) Matros [53]	Brigham	103	2	KRT20
8) Ben [54]	Shanghai	94	2	L1CAM
9) Livosky [55]	MGH	91	1	LLGL2
10) Coppola [56]	South Florida	82	1	SH3GLB1
11) Yang [57]	Xi’an	78	1	PSCA
12) Chung [58]	Yale	76	3	FLT1
13) Tong [59]	MDA	73	1	LCN2
14) Cates [60]	Vanderbilt	68	3	TWIST1
15) Cantile [61]	Naples	64	1	HOXD13
16) Marsh [62]	Ohio State	56	11	CNN1
17) Yang [63]	Xi’an	51	1	S100A6
18) Morse [64]	MCC	42	2	ABCC3 and TLR2
19) Gray [65]	ACC	35	1	PLK1
20) Wen [66]	Yonsei	31	2	POU5F1 and NANOG
21) Pham [67]	Toronto	26	18	PTEN and STAT3

Pubmed search: tissue[Title/Abstract] AND microarray[Title/Abstract] AND pancreas and cancer.

Abbs: JHH (Johns Hopkins Hospital), MDA (MD Anderson), MCC (Moffitt Cancer Center), ACC (Arizona Cancer Center), Abs (antibodies).

* The present study is the only one that compared patients with short and long survival.

### Selection of Biomarkers

A published compendium of putative pancreatic cancer biomarkers based on a comprehensive literature search lists 2,516 overexpressed genes (∼10% of the genome) in pancreatic cancer [22]. Due to the overwhelming number of candidate biomarkers, we designed a strategy to select a panel of antibodies for use against the s-TMA (Figure 1). We pared the list of possibilities down to 386 candidate genes previously studied using IHC. The list of IHC biomarkers in PDA was then cross-referenced with our institutional pathology catalogue of 380 optimized antibodies. Our pathology core contained 65 optimized antibodies against putative PDA biomarkers. Due to the finite number of unstained histologic sections available from a TMA for study (∼50 in the present resource), we further refined the selection process by stratifying biomarkers according to the number of independently published IHC studies cited in the central biomarker repository. Using this strategy, 13 PDA biomarkers were identified as the subject of four or more IHC-based peer-reviewed studies, and additionally were already optimized in our pathology core (BCL2, CASP3, CCND1, EGFR, ERBB2, MSLN, MUC1, MUC4, P53,SMAD4, MUC5AC, BIRC5, and ITGB4). The first 10 were selected for testing in the present study. In addition, three antibodies were included as representative samples from the remaining choices of putative PDA biomarkers: CEACAM6 (2 previous IHC-based publications), MYC (2 publications), and MUC2 (1 publication). MUC2 was believed to be particularly intriguing due to its association with indolent pancreatic tumors, in direct contrast to MUC1, which has been linked with more aggressive pancreatic tumor subtypes [25], [26].

### Immunohistochemical Analysis

Immunohistochemical analyses were performed by a standard streptavidin-biotin-peroxidase procedure. Labeled TMA sections were subjected to heat-induced epitope retrieval with the Ventana Discovery XT automated system (Ventana Medical Systems, Tucson, AZ). Primary antibodies and their dilutions included: BCL2 (1∶100, DAKO, Carpenteria, CA), CASP3 (1∶300), CCND1 (1∶25, Lab Vision, Fremont, CA), CEACAM6 (1∶5, Biogenex, San Ramon, CA), EGFR (1∶100, Zymed, Carlsbad, CA), ERBB2 (1∶400, Signet, Princeton, NJ), MSLN (1∶100, Vector Labs, Burlingame, CA), MUC1 (1∶100, Vector Labs, Burlingame, CA) [27], MUC2 (1∶100 Vector Labs, Burlingame, CA) [27], MUC4 (1∶3000, clone 8G7, a gift from University of Nebraska) [28], MYC (1∶2000, Epitomics, Burlingame, CA), P53 (1∶500, DAKO, Carpenteria, CA), and SMAD4 (1∶800, Santa Cruz Bio, Santa Cruz, CA). The Ventana DABMap Kit was used for antibody detection.

Immunohistochemical review was performed by an expert pancreatic pathologist (L.H.T.) and recorded by a different study investigator (J.M.W). SMAD4, MUC2, and BCL2 were scored as negative or positive by IHC based on previous scoring strategies [29]. For all other antibodies, a 4-point scale (from 0 to 3+) was applied based on the percentage of labeled cancer cells in the tissue core: 0 (<10% labeled cells), 1+ (11–25%), 2+ (26%–75%), 3+ (>75%). An average score was recorded for each triplicate set. A total of 14 samples had insufficient neoplastic cellularity for IHC analysis and were excluded, yielding 137 samples with adequate tissue for all tested antibodies.

### Statistical Analysis

The analysis was performed using Intercooled Stata 8.2. Categorical variables were tested by the Fisher’s exact test, continuous variables by the Wilcoxon rank sum test, and standard logistic regression was performed for multivariate testing. Continuous variables were tested using the rank sum test. A comparison of multivariate regression models was performed to identify the best prognostic model using receiver operating characteristic analysis and the associated Harrel’s C-index (also referred to as area under the curve or ROC area). In the present analysis, the C-index measures how well a particular multivariate model of predictors discriminates between short- and long-term survival groups. The values ranged between 0 and 1. A value of 0.5 indicates no predictive ability (random prediction) and appears as a diagonal line on an ROC graph, whereas values above 0.5 indicate good predictability, and appear as curvilinear plots above the diagonal. When two ROC curves do not intersect, the one with a higher C-index dominates over the other. All statistics were two-tailed with a p value <0.05 indicating statistical significance.

## Results

### Conventional Pathologic Features

There were 79 (58%) patients in the long-term survivor group and 58 (42%) in the short-term survivor group. Conventional pathologic features (lymph node status, histologic grade, size, and resection margin status) and patient variables (age, gender, postoperative length of stay, and adjuvant treatment) were analyzed as predictors of survival group (Table 1). None of the patient-related factors correlated with survival. Of the pathologic variables, positive lymph node status (p = 0.02), poor differentiation (p = 0.003), and a tumor size greater than 3 cm (p = 0.02) were associated with early cancer-specific death in the unadjusted univariate analysis.

The prognostic accuracy of three different models, as estimated by the Harrell’s C-index, was compared, graphed and tabulated (Figure 4). The model that included the three biomarkers (MUC1, MSLN, and MUC2) was superior to the model including four conventional pathologic features (lymph node status, histologic grade, tumor size, and resection margin status), although the difference just missed statistical significance (p = 0.07). The combined model with biomarkers and pathologic features performed the best (p = 0.0001).

### Biomarkers

Expression patterns of the 13 candidate prognostic markers (see the Methods and Figure 1 for details on biomarker selection strategy) in the two survival groups were tested and compared. The univariate results are provided in Table 2. Out of 13 candidate genes, only MUC1, MSLN, and MUC2 had statistically different expression patterns between groups. A trend towards significance was observed with SMAD4 loss (p = 0.09). Representative slides labeled with MUC1, MSLN, and MUC2 appear in Figure 2.

### MUC1

A strong association was observed between increased MUC1 protein expression and short survival (p<0.0001). In the total cohort, 15% of patients had an IHC score of 0, 23% had 1+, 33% had 2+, and 30% had 3+. The proportions of patients that were in the short survivor group at each separate IHC score increased in a linear fashion (slope of linear regression  = 0.21, p = 0.002). Specifically, 5% were in the short survival group with an IHC score of 0, 29% with 1+, 49% with 2+, and 68% with 3+ (Figure 3A). The negative predictive value was high (95%), as 19 out of 20 patients with absent MUC1 expression in this cohort survived more than 30 months.

### MSLN

As compared to MUC1 expression, the pattern of MSLN expression in the total cohort was slightly weighted towards lower IHC scores: 63% of patients had 2+ or 3+ MUC1 labeling while 48% had comparable MSLN labeling (p = 0.02). However, like MUC1, there was a strong association between MSLN expression and early cancer-specific mortality (p<0.0001). Again, a linear relationship was observed between the IHC score and the proportion of patients in the poor survival group (slope of linear regression  = 0.20, p = 0.02). In the different IHC score categories, the percentage of patients that were in the short survival group were as follows: 20% of the patients with an IHC score of 0, 35% with 1+, 49% with 2+, and 81% with 3+ (Figure 3B).

### MUC2

MUC2 expression was associated with long survival in contrast to MUC1 and MSLN (p = 0.03). MUC2 expression was uncommon overall (15%) in PDA. Short-term survivors expressed MUC2 in just 7% of cases. Long-term survivors expressed MUC2 in a greater proportion, although expression was still uncommon (22% of cases).

### Multivariate Analysis

A multivariate logistic regression analysis was performed which included significant univariate biomarker predictors of survival (MUC1, MSLN, and MUC2) as well as the four commonly reported pathologic features (Table 3). MUC1 and MSLN were highly significant in the adjusted model, while MUC2 was not. As compared to absent expression, odds ratios associated with incremental MSLN expression were 1.7 (IHC score  = 1+), 2.6 (IHC score  = 2+) and 12.5 (IHC score  = 3+). For MUC1, the odds ratios were 10.1 (IHC score  = 1+), 11.9 (IHC score  = 2+) and 29.0 (IHC score  = 3+). The composite p-values for MSLN and MUC1 were p = 0.01 and p = 0.004, respectively. None of the conventional pathologic features were statistically significant in the multivariate model. To test whether the high prognostic values of MUC1 and MSLN were merely an artifact of a multi-tiered comparison (IHC scores of 0 to 3+), the multivariate model was repeated after categorizing lymph node metastases in a similarly tiered fashion (negative, 1, or ≥2 lymph node metastases). Adjusted odds ratios for the relevant biomarkers were unchanged; multiple lymph node metastases predicted poor survival with an odds ratio of only 3.9 (p = 0.02).

## Discussion

Early cancer recurrence and mortality after pancreatic resection (within one year) remain disheartening experiences for clinicians. In these instances, patients with seemingly “resectable” disease have major resections with “curative intent,” yet do not receive any definable benefit, occasionally at the cost of significant morbidity or even mortality. Our institutional data suggests this scenario occurs in one–fifth of patients who undergo pancreatic resection for PDA [30]. On the other hand, some patients with metastatic disease have relatively slow growing cancers, and might benefit from metastasectomy or cytologic reduction. This scenario is extremely uncommon with PDA, yet there is precedent for an aggressive surgical approach in selected patients with advanced but indolent disease [31]. Unfortunately, the present approach to patients with PDA fails to integrate biologic factors. At the present time, conventional pathologic features provide the best prognostic information, yet are not sufficiently reliable to impact treatment decisions, as the present study shows.

Studies designed to identify reliable prognostic markers face two particular challenges. First, extrinsic determinants of survival which are independent of a tumor’s molecular profile confound biomarker analyses. Consider a scenario in which the difference in overall survival between two patients after pancreatic resection is only 3 months. The survival difference may be related to patient performance status, social factors, medical comorbidities, chemotherapy response, treatment toxicity, surgical complications, or a number of other possibilities. Each of these factors may minimally contribute to patient survival, and would require a study with very large statistical power to fully characterize each one. Furthermore, these survival factors are not typically associated with biomarker expression patterns (chemotherapy response and toxicity may be exceptions). The present study minimizes noise from alternative and less significant survival factors by excluding patients with intermediate survival (12–30 months). Except for rare instances, tumor biology would be expected to be the principal driver of survival groups defined by a time gap of this magnitude (a minimum of 1.5 years separates short and long survivor groups).

We identified 20 other studies in the literature that analyzed protein biomarkers using TMAs of PDA (Table 4). Unlike the present study, these TMA-based studies included all patients across the survival spectrum, which may be interpreted as a positive study feature. None of these studies identified any biomarkers with clinical relevance in PDA. We suggest that a survival TMA may be better suited for biomarker discovery investigations in PDA, because it emphasizes tumor biology. The sample size in the present study compares favorably with other TMA studies (top quartile). Most important, this study likely includes the largest number of patients at the survival extremes.

The second challenge for biomarker surveys of PDA with IHC is to devise a rational strategy to select the best molecular candidates for study. There are roughly 30,000 human proteins, and 10% have been reported as overexpressed in PDA [22]. IHC analyses are limited by the amount of available tissue (one antibody per TMA section, and roughly 30–50 sections per TMA block), and therefore a rational candidate biomarker selection process is required to select the most practical and promising biomarkers for study. Typically, investigators design experimental biomarker panels according to either research interests, an intriguing paper, or a unifying theme such as a common molecular pathway. As Table 4 illustrates, previous TMA studies in PDA test a small number of antibodies (median of 2 biomarkers per study; range, 1 to 18). Only two studies examined more than 4 antibodies.

Biomarker selection in the present study was based on the recently published and centralized biomarker repository for PDA [22]. Construction of this dataset was a massive effort which required 7000 person hours (amounting to nearly one person’s work per year). The authors identified every study in the literature that linked a gene or protein to PDA, and then tabulated the index gene (or protein), the principal assays involved in the study, and the relevant reference. The authors’ primary goal was to “develop a compendium of potential biomarkers that could be systematically validated by the pancreatic cancer community” [22]. Putting their challenge to action, we analyzed this large dataset using an algorithm (detailed in Figure 1) [22] that placed increased importance or weight on the number of previous reports linking a given biomarker to PDA. A total of 13 biomarkers were identified using this selection strategy and corresponding antibodies were tested against the s-TMA.

IHC analysis revealed that 10 of the13 candidates were non-informative as prognostic markers in this study cohort. These negative observations provide convincing evidence (with the exception of SMAD4 which missed statistical significance, p = 0.09) that this group of putative pancreatic cancer biomarkers are clinically irrelevant for prognosis. In the univariate analysis, MUC1 and MSLN expression were associated with aggressive cancer biology (i.e. short survival group) and MUC2 expression was associated with favorable biology (i.e. long survival group). Only MUC1 and MSLN were robust prognostic factors in the multivariate model, adjusting for conventional pathologic features (Table 3). Diffuse MUC1 and MSLN expression were highly predictive of short survival (the odds ratios were 12.47 and 28.95, respectively). Interestingly, the four standard pathology tests had odds ratios below 3, and none achieved statistical significance in the multivariate model. An ROC analysis was performed to estimate the predictive accuracy of three different multivariate models at distinguishing short and long survival groups (biomarkers only; pathologic features only; and a combination of biomarkers and pathologic features). A trend towards superior predictive accuracy was observed with the panel of molecular markers (MUC1, MSLN, and MUC2) over conventional pathologic features (AUC = 0.81 vs. 0.70, p = 0.07). These data suggest that biomarkers may actually provide more prognostic insight than standard prognostic data included in pathology reports.

This study validates MUC1 and MSLN as biomarkers of aggressive pancreatic cancer biology. The implications of these findings must be interpreted in the context of the study design, and the role of these proteins as prognostic markers in the clinical management of PDA remains uncertain. While each oncoprotein has been the focus of over 200 studies in PDA, there are no large-scale studies of tumor samples that have thoroughly examined them as prognostic biomarkers using a comparable graded IHC scoring system. Some previous studies have observed survival differences associated with high and low expressing tumors (MUC1 or MSLN), but are limited by small sample sizes, the absence of tiered IHC scoring systems, and unadjusted statistics [32]–[34].

Certain biomarkers included in the study had expression patterns that differed from previous reports. For instance, MSLN expression (1+ or greater) was observed in 71% of patients with PDA in the present study (as compared to 85–100% in prior studies [32], [35]) and MUC4 expression (1+ or greater) was observed in 55% (as compared to 90% in prior studies [36], [37]). Differences between this study and previous ones may be related to sample size variability (previous studies were smaller) and patient selection (the present study is enriched with patients at the survival extremes). We are in the process of validating the results of the present study in a large dataset that includes patients across the entire survival spectrum. In addition, differences in immunohistochemical scoring are important. For instance, previous studies of MSLN and MUC4 defined positive labeling as focal antibody reactivity in more than 1% of cancer cells [35], [36], while the present study required at least 10% of cells for an IHC score of 1+.

The implications of this research extend beyond improved prognostic assessment of tumor samples, and therefore the utility of the s-TMA strategy is not entirely contingent on validation studies with large numbers of unselected patients. First, biomarker discovery based on survival extremes is useful to prioritize cancer genes for diagnostic and therapeutic research. Since the most biologically aggressive cancer cells are typically refractory to conventional agents, it stands to reason that novel treatment approaches that specifically target aggressive sub-clones are particularly appealing and warrant further investigation. In support of this concept, the NCI has identified MUC1 and MSLN among the most promising targets for cancer vaccine development, with the former protein listed in the top three [38]. A radiolabeled monoclonal antibody against MUC1 was also recently evaluated in a phase I/II trial, with a planned phase III trial to follow [39], [40]. Furthermore, promoter-driven cancer gene therapy which exploits overactive MUC1 and MSLN promoters in various cancer types has been extensively studied in pre-clinical cancer models using viral vectors [41]–[43]. We are currently pursuing a promoter-driven gene therapy approach against PDA using a non-viral, biodegradable polymer vector to deliver toxic nanoparticles [16].

Additionally, both MUC1 and MSLN are present on the cell-surface with secreted isoforms. Thus, prognostic markers such as these are potentially detectable in sera or secreted fluids. Reliable noninvasive tests that correlate with membrane-bound isoforms may function as surrogate biomarkers to biologically stratify patients or perhaps select them for targeted therapies. An FDA approved ELISA test of soluble mesothelin-related proteins (Mesomark® Assay, Fujirebio Diagnostics, Malvern, PA) holds great promise as a serum and pleural fluid marker for malignant pleural mesothelioma [44]. The Mesomark Assay has been evaluated in a single study of PD; the study was not powered to test prognostic capability and did not compare levels with tumor MSLN expression [45]. No studies have examined the prognostic potential of soluble-MUC1 in PDA.

We are presently evaluating additional candidate prognostic markers against the s-TMA to further optimize the predictive model. Based on the strategy used to select candidate markers in the present study, additional intriguing proteins include MUC5AC, BIRC5, and ITGB4. A high-throughput proteomic or transcriptomic analysis of survival extremes could identify novel prognostic markers, but is best suited for tumor samples enriched for neoplastic cells such as tumor cell lines or xenografts (as opposed to primary tumor tissue such as the samples in this study with abundant stroma) [46]. A high-throughput molecular analytic strategy would obviate the need for a pre-assay biomarker selection process for candidate immunohistochemical markers such as the one described in Figure 1. The disadvantage is that the results reflect the molecular profile of a clonal cancer cell population derived from the original tumor (and likely a particularly aggressive clone selected for under laboratory conditions), which may not reflect the biology of the rest of the tumor. Gene expression analyses comparable to the Oncotype Dx® for breast cancer [18], would likely require very large numbers of primary tumor samples to determine an effective prognostic panel, particularly because of less biologic heterogeneity with PDA.

### Conclusions

This study presents the results of a survival-based TMA (s-TMA), comprised of patient tumor samples associated with short and long-term survival after resection for PDA. The s-TMA was used to identify bona fide protein markers of aggressive tumor biology. MSLN and MUC1 were highly significant predictors of early cancer-specific mortality, and were superior to conventional pathologic features as prognostic markers.
