# Correction: A Novel Survival-Based Tissue Microarray of Pancreatic Cancer Validates MUC1 and Mesothelin as Biomarkers

**DOI:** 10.1371/annotation/2533f354-bbec-404a-9661-2c052963b918

**Published:** 2012-08-07

**Authors:** Jordan M. Winter, Laura H. Tang, David S. Klimstra, Murray F. Brennan, Jonathan R. Brody, Flavio G. Rocha, Xiaoyu Jia, Li-Xuan Qin, Michael I. D’Angelica, Ronald P. DeMatteo, Yuman Fong, William R. Jarnagin, Eileen M. O’Reilly, Peter J. Allen

The affiliation for the first author is incorrect. Jordan M. Winter is not affiliated with #1 but with #3 Department of Surgery, Memorial Sloan-Kettering Cancer Center, New York, New York, United States of America.

Jordan M. Winter's current address was omitted. It is Department of Surgery, Philadelphia, Thomas Jefferson University, Pennsylvania, United States of America 

